# Emergence of Rice Blast *AVR-Pi9* Resistance Breaking Haplotypes in Yunnan Province, China

**DOI:** 10.3390/life13061320

**Published:** 2023-06-04

**Authors:** Lin Lu, Qun Wang, Zhufeng Shi, Chengyun Li, Zhixiang Guo, Jinbin Li

**Affiliations:** 1 Flower Research Institute, Yunnan Academy of Agricultural Sciences, Kunming 650205, China; 2 Yunnan Key Laboratory of Green Prevention and Control of Agricultural Transboundary Pests, Agricultural Environment and Resource Research Institute, Yunnan Academy of Agricultural Sciences, Kunming 650205, China; 3 The Ministry of Education Key Laboratory for Agricultural Biodiversity and Pest Management, Yunnan Agricultural University, Kunming 650200, China

**Keywords:** rice blast, virulence, polymorphism, monogenic resistance, landrace

## Abstract

The rice blast disease (caused by *Magnaporthe oryzae*) is a devastating disease in China. Understanding the molecular mechanisms of interaction for the cognate avirulence (*AVR*) gene with host resistance (*R*) genes, as well as their genetic evolution is essential for sustainable rice production. In the present study, we conducted a high-throughput nucleotide sequence polymorphism analysis of the *AVR-Pi9* gene that was amplified from the rice-growing regions of the Yunnan Province in China. We detected the presence of seven novel haplotypes from 326 rice samples. In addition, the sequences of *AVR-Pi9* were also obtained from two non-rice hosts, *Eleusine coracana* and *Eleusine indica*. The sequence analysis revealed the insertions and deletions in the coding and non-coding regions of the gene. The pathogenicity experiments of these haplotypes on previously characterized monogenic lines showed that the newly identified haplotypes are virulent in nature. The breakdown of resistance was attributed to the development of new haplotypes. Our results suggest that the mutation in the *AVR-Pi9* gene is an alarming situation in the Yunnan province and thus needs attention.

## 1. Introduction

About 50% of the world’s population uses rice as a staple food. Increasing rice production is the ultimate goal for meeting the demands of the ever-increasing world population. Although several rice varieties have been introduced over the years, high-yielding cultivars are, ironically, continuously threatened by biotic and abiotic stresses. The fungal pathogen that causes the blast disease (*Magnaporthe oryzae*) is the most serious worldwide source of disease for rice. It infects several cereal crops such as rice, wheat and rye grass [1,2]. Its infection can cause up to a 30% annual loss in terms of rice grain yield [3]. Studies have shown that the initial infection of the fungus is biotrophic; its hyphae invade and penetrate the plant cells [4]. In later stages, the fungus becomes necrotic and causes the death of the host cells [5]. Rice and its blast disease fungus represent the best pathosystem through which to understand the dynamics of the host–pathogen interaction [6]. Resistance to *M. oryzae* is controlled by resistance genes (*R* genes) in rice [7]. It is well known that the avirulence (*AVR*) genes in *M. oryzae* correspond to the *R* genes in rice [8]. Historically, the continuous selection pressure on hosts and their fungal pathogens has led to co-evolution. The selection of *R* genes and the mutation of pathogen-encoded *AVR* genes are considered the main driving forces in the arms race hypothesis. There is a continual consensus that the interaction between *R* and *AVR* genes lead to their diversification [9]. Previously, a *Japonica* rice variety, Lijiangxintuanheigu (LTH), was introduced in Yunnan Province [10,11]. LTH is a well-known susceptible variety to thousands of isolates of *M. oryzae*. Several *R* genes such as pi60, pi61, pi-g, pi36 and pi19 were cloned based on the genome sequence information obtained from LTH cultivars [12]. In addition, near-isogenic lines that contained single Pi genes were developed to assess the genetic structure and to monitor the new pathotypes of *M. oryzae.*

The study of *R-AVR* gene interactions suggests three different modes of host–pathogen interactions [13]. In the first mode, a single *R* gene directly interacts with its corresponding *AVR* gene [14]. The second type is characterized by the interaction of one *R* gene with two unrelated *AVR* genes [15], while the third type of interaction is represented by the fact that different *R* genes may interact with different *AVR* genes. The blast resistance developed by conventional breeding is overcome due to the high genetic diversity of *AVR* genes [16].

The *Pi9* locus in indica rice shows a broad spectrum resistance to a wide range of *M. oryzae* strains [17]. It is predicted that *Pi9* is expressed at high levels in the rice plants and contains a cluster of NBS-LRR [18]. The *Pi9*-*R*-gene-based resistance strategy is effective against *M. oryzae* due to the presence of corresponding *AVR*-*Pi9* genes, which can trigger the plant defense mechanism. *AVR*-*Pi9* is a small gene that codes for only 99 amino acids and is located on chromosome 7 of the *M. oryzae* genome [19]. The corresponding *Pi9* is thought to have been introduced from *Oryza minuta* [19]. The *Pi9* monogenic lines, developed at the International Rice Research Institute (IRRI), show resistance to most rice blast strains, except IE1k [16].

The higher genetic variability in *M. oryzae* avirulent genes is considered to be an important factor in defeating the *R* genes encoded by plants. Recently, a diversity analysis of *AVR* genes from 60 rice blast strains was conducted in Thailand. Only a single amino acid change was detected in the *AVR-Pi9* gene. The data suggested that *AVR-Pi9* has relatively less genetic diversity compared to other *AVR* genes [20]. 

The present study was aimed to demonstrate the genetic diversity and distribution of the *AVR-Pi9* gene in *M.-oryzae*-infected rice and non-rice plants in Yunnan province, China. We collected 328 samples of the blast fungus, and the *AVR-Pi9* gene was amplified using specific primers. A sequence comparison indicated the presence of seven different mutants. In this study, the pathogenicity and dynamics of rice blast fungus are discussed. 

## 2. Materials and Methods

Source, isolation and culture of fungi: About 326 isolates of *M. oryzae* and 2 non-rice fungi were collected from Yunnan Province, China. The samples were collected based on the manifested disease phenotypes in rice plants [21,22,23]. The in planta fungal isolates were collected from the infected rice plants and observed under a light microscope. The isolates were transferred to filter paper at freezing temperatures until they were grown on oatmeal-supplemented agar. All the spores were grown at room temperature under controlled lighting [24]. After confirmation of *M. oryzae*, the fungal spores were isolated and cultured in the dark at 25 °C for 7 days, as was previously described in [25,26].

DNA isolation, PCR amplification and sequencing: To produce mycelium, the fungal isolates were grown in the dark at 25 °C for 6–8 days. The total genomic DNA was isolated via grown mycelium and plant leaves while using the cetyl trimethylammonium bromide (CTAB) method, as was described previously in [27,28]. The isolated DNA was run on 1% agarose gel and quantified using a spectrophotometer (Nanodrop 2000). Approximately, 50 ng/µL of DNA was used in the PCR amplification reaction. Each PCR was performed in triplicates. Briefly, full-length *AVR-Pi9* specific primers were used in a standard 25 µL PCR reaction. *AVR-Pi9F* atgcagttctctcagatcctcacc and *AVR-Pi9R* ctaccagtgcgtcttttcgacttg [19,29] were synthesized by Tiangen Biotech Co., Ltd., Beijing, China. All the PCR products were resolved on 1% agarose gel, and the amplification of ~1500 nucleotides (nts) was confirmed (DL2000 DNA Ladder; Tiangen Biotech Co., Ltd., Beijing, China). All the PCR products were sequenced by the specific primers (Shanghai Life Technologies Biotechnology Co., Ltd., Beijing, China).

Sequence assembly and nucleotide diversity analysis: The *AVR-Pi9* sequence data were annotated in the SeqMan module of Lasergene-7 (DNA-STAR, Madison, WI, USA). The accurate small reads of ~800 nts were assembled into a single consensus file. To validate the sequencing, the sequences obtained from different isolates were compared, via BLASTn, with the reference sequences already available in the NCBI GenBank. The *AVR* sequence files of 328 (326 rice and 2 non-rice) isolates were aligned using Clustal-X [30]. For the phylogenetic analysis, the aligned sequence files were exported to MEGA 11.0 software [31]. The phylogenetic tree was constructed using the maximum likelihood method of tree construction with 1000 bootstrap replications. The nucleotide polymorphism, haplotype diversity, (*π* and *H_d_*_,_ respectively), and DNA haplotype polymorphic sites were calculated using the DnaSP-6 software package [32,33,34]. Population neutrality and selection pressure tests were calculated using Tajima’s D test [35,36]. The DNA sequence-based TCS software was used (http://darwin.uvigo.es/software/tcs.html, accessed on 25 September 2022) to estimate the gene genealogies and haplotype networks.

Disease pathogenicity assay: The pathogenicity test was performed on the resistant genotype (IRBL9-W) that harbors the *Pi9* gene in the background of LTH. The LTH lines were used as a susceptible control [37]. Briefly, the conidia were randomly collected and maintained on a culture medium, as was described previously in [29,38]. The rice seedlings were grown for 21 days, and the spore suspension that contained conidia was sprayed on the leaves. The spore suspensions of these isolates (10^5^ spores/mL) were sprayed on 3–4 leaf stage seedlings. After inoculation, the plants were transferred to a dark room with more than 90% humidity at 25 °C for 24 h. After 7 days, the plants were photographed and disease symptoms were scored according to a previously described scale [24]. 

## 3. Results

### 3.1. Distribution of M. oryzae Isolates in Yunnan Province, China

A total of 328 samples were collected from the Yunnan province of China during the growing seasons of 2010 to 2020 (Table 1). The main sampling area included the central, northern, southern and eastern areas of the province. In the PCR reaction with specific primers of *AVR-Pi9* genes, we obtained a positive amplification in 328 samples (Table 1). To exclude possible contamination, each PCR was repeated three times. The sequencing results also confirmed the presence of *M. oryzae* in all the samples collected.

The pathogenicity assay indicated that all the isolates were avirulent to the monogenic IRBL9-W line. The frequency of the virulent strains was 100% in the field, with the exception of the western region—where it was observed as 96%. Overall, the frequency of the *AVR-Pi9* gene was recorded as 98% (Table 1). The nucleotide alignment of the *AVR-Pi9* gene revealed the presence of seven different haplotypes (H1–H7). The nucleotide variation analysis indicated that minor mutations were scattered in the promoter, coding and intronic regions. These findings suggested that although *Pi9* is a well-known source of resistance to *M. oryzae*, virulent strains are also circulating in the region, which may lead to resistance breakdown on a large scale (Table 2).

### 3.2. Identification of AVR-Pi9 Haplotypes

**Figure 1:**
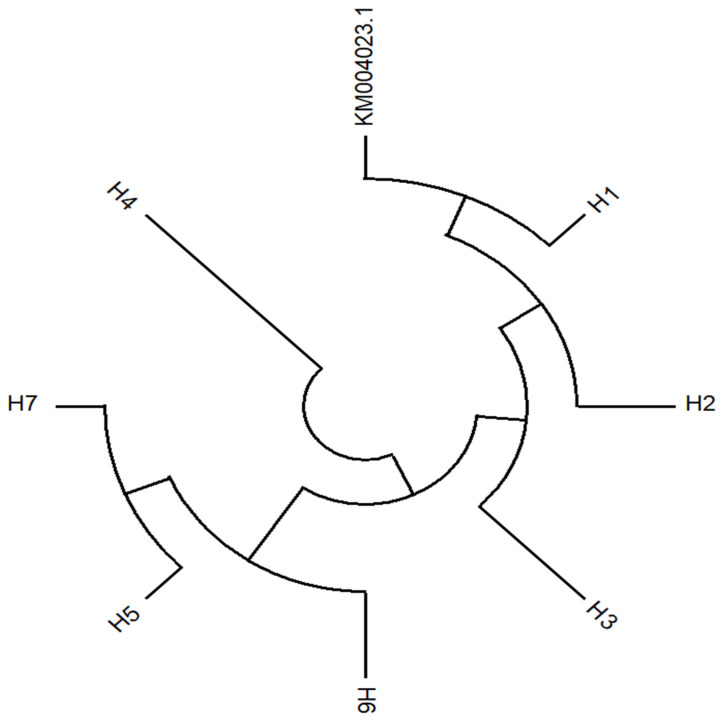
Maximum likelihood phylogenetic tree of the seven haplotypes identified from rice in the Yunnan province of China. The seven haplotypes can be divided into three different clusters as per their sequence variation. The H1 haplotype showed high genetic identity with the reference strain (KM004023.1).

**Figure 2:**
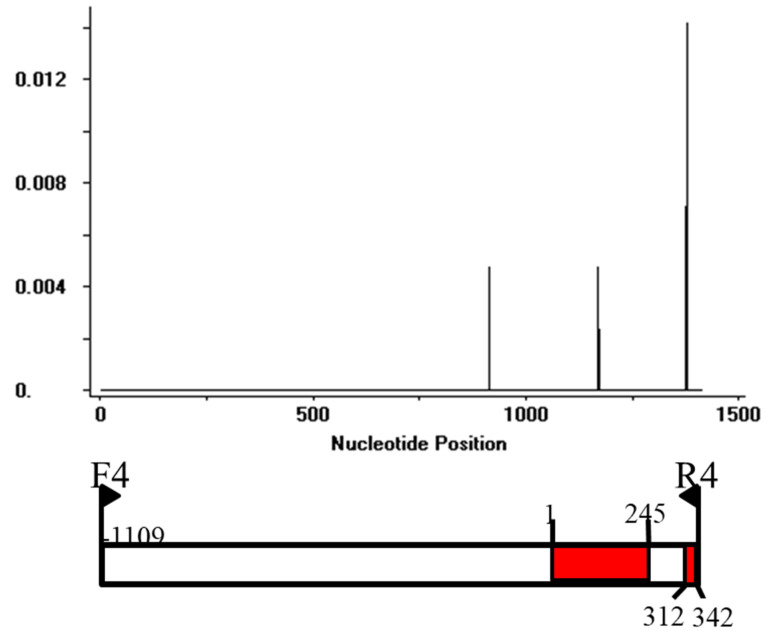
The diversification of *AVR-Pi9* in avirulent isolates. The distribution of variation in the *AVR-Pi9* alleles was analyzed via the sliding window method. The *X*-axis shows the distribution of variation within the full region, including the promoter region and exons of *AVR-Pi9*. The lower pane indicates the corresponding schematic presentation of the promoter region in white color, and two exons in red color are for *AVR-Pi9*. Window length: 10. Step size: 2. The π value corresponds with the level of variation at each site because it is the sum of the pair-wise differences divided by the number of pairs within the population.

The *AVR-Pi9* gene consisting of 1436 nucleotides was amplified from the 328 isolates by using gene specific primers. Amplified PCR products from 328 isolates (including 326 isolates collected from rice and 2 isolates collected from non-rice) were sequenced and assembled. The gene sequence was compared with the KM004023.1 reference sequence (Table 3). After the sequence annotation and assembly analysis, eight different haplotypes (H1–H8) were identified (Table 3). H1 was identified as the major haplotype with the highest percentage (66.5%) among the other seven haplotypes (Table 3, Figure S1). Sequence analysis revealed the highest sequence identity of the CDS region in the reference sequence. The H2 haplotype was characterized by a deletion of 15 nucleotides at the variant locus. Interestingly, H2, H3 and H4 haplotypes were characterized as deletion mutants in the promotor region of the variant locus. In addition, the H3 haplotype has a substitution (T-nucleotide) when compared to the reference strain. The H5, H6 and H7 haplotypes were found at very low frequencies. However, these haplotypes showed nucleotide substitutions in the CDS region (Table 3). The sequence variation of these seven haplotypes was also evident from the phylogenetic tree (Figure 1). The maximum likelihood phylogenetic tree showed three major groups. One group contained the reference sequence, H1, H2 and H3 haplotypes. Haplotype H4 diverged due to sequence variation in the promoter region (Table 3). The haplotypes H5, H6 and H7 were clustered together due to high-nucleotide identity. However, there were differences in the positions of the nucleotide substitution sites among these three isolates. H8 haplotype contained two isolates collected from *Eleusine coracana* and *Eleusine indica*, respectively. The diversity analysis of the haplotypes suggests that *AVR-Pi9* may be under either a strong population expansion or a purifying selection due to the introduction of resistant rice germplasms. The results of the polymorphic analysis show that *AVR-Pi9* may be under a strong positive selection. The sliding window for the distribution of Ka/Ks values suggests the possibility of potential purifying positive selections (Figure 2). Indeed, the sites of positive selection were clearly present in the exon region of the *AVR-Pi9* gene. The haplotype diversity and polymorphic analysis (π) also confirmed the distribution of variation in the CDS region (Figure 2). 

### 3.3. Genetic Variation of the AVR-Pi9 Gene in Non-Rice Hosts

In this study, the *AVR-Pi9* gene from three non-rice hosts, including perennial rye grass and wheat (Table 4), is investigated. The *AVR-Pi9* sequence from *Setaria viridis* was also included in this study and was obtained from GenBank. Notably, the non-rice isolates had high genetic similarity to the previously known sources of the *AVR-Pi9* gene. Sequence comparison showed that the isolates from ryegrass and Setaria were 98% identical to the reference sequence (*AVR-Pi9*-KM004023), while the two isolates from wheat, namely PY5033 and PY6045, showed a 97% sequence identity with the reference strain. Close examination revealed the deletions in the nucleotide sequence of the non-rice hosts. 

### 3.4. Selection Pressure of AVR-Pi9 in M. oryzae

To determine the natural selection pressure of the *AVR-Pi9* in the *M*. *oryzae* from Yunnan Province, the Tajima’s neutrality of the *AVR-Pi9* in *M*. *oryzae* was tested based on 328 *AVR-Pi9* DNA sequences, and the Tajima’s *D* was −1.85719 (Table 5). The lower value of Tajima’s D indicates the high number of alleles in the rice blast population in Yunnan Province, China. These results also suggest that *AVR-Pi9* may be under either strong population expansion or purifying selection due to the introduction of resistant rice germplasms.

### 3.5. The Phylogenetic Relationship of AVR-Pi9 Haplotypes

**Figure 3:**
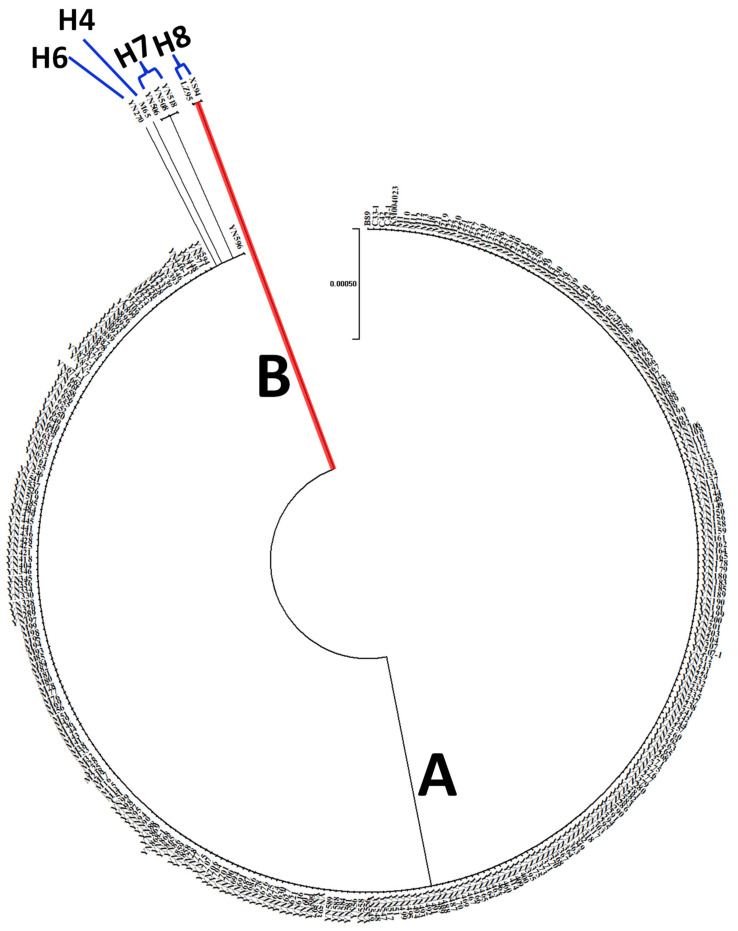
The phylogenetic tree constructed with the nucleotide sequences of the *AVR-Pi9* genes from 328 *Magnaporthe oryzae* isolates. The maximum likelihood methods of MEGA V5.10. The ID number of KM004023 (GenBank Accession No. KM004023) for *AVR-Pi9* was obtained from GenBank.

To understand the relationship of isolates from the Yunnan province, the phylogenetic tree was constructed from the amplified fragment of *AVR-Pi9*. The phylogenetic tree that was based on the *AVR-Pi9* sequences of 328-blast isolates showed that the majority of them were closely clustered, except H4, H6 and H7 (Figure 3). The close clustering of isolates confirmed their low level of genetic diversity. However, smaller but significant changes in the H4, H6 and H7 isolates resulted in their unique position in the phylogenetic tree. Among the two clusters (A and B), Cluster A included only the isolates collected from rice, while Cluster B contained the isolates from the *Eleusine coracana* and *Eleusine indica* of the H8 haplotype.

### 3.6. Pathogenicity Assay

**Figure 4:**
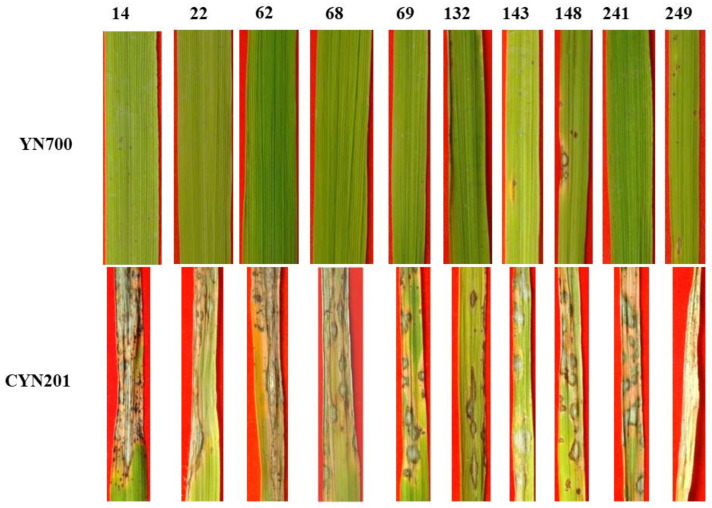
Disease reaction of the identification isolates of YN700 and YNC201 on the landrace accessions. YN700 (which belongs to the H1 haplotype) and YNC201 are avirulent and virulent to IRBL9-W (holding *Pi9*), respectively. Note: the numbers 14, 22, 62, 68, 69, 132, 143, 148, 241, 249 on top of the photo show the landrace varieties of Qi-He-Da-Hong-Gu, Lao-Leng-Gu, Da-Huang-Pi-Nuo, Bei-Zi-Nuo, Zao-Hong-Gu, Xiao-Hua-Gu, Da-Bo-Gu, Hao-Bu-Ka, Qie-Xie-Gu and San-Bai-Bang, respectively.

The previous data showed that the rice varieties harboring the *Pi9* gene showed broad-spectrum resistance to *M. oryzae*. We tested the disease response of the existing rice blast fungus on the landraces collected from Yunnan Province, China. The landraces that were tested against YN700 (H1 haplotype, identical to haplotype KM004023.1) were resistant and showed avirulence. This test confirmed the existing hypothesis that monogenic lines carrying the *Pi9* gene are resistant to blast fungus. To confirm the resistance and susceptibility, we inoculated the landraces with a rice blast fungus containing the *AVR-Pi9* allele. In the experiment where the haplotype KM004023.1 was used, none of the landraces developed a positive disease response (Figure 4). All the previously tested landraces consistently showed resistance to the existing haplotypes. Nonetheless, the inoculation of CYN201 (H8 isolated from rye grass) on the same ten landraces resulted in a sever disease reaction. All landraces showed sever blast symptoms and eventually died. These data led to the conclusion that the newly identified haplotypes may be lethal to rice production in Yunnan Province, China.

## 4. Discussion

The introgression of *R* genes into rice germplasm was an important effort to conduct in order to regain the broad-spectrum resistance of *Magnaporthe oryzae* [16]. Several monogenic and digenic lines have been developed worldwide to counter the damage caused by rice blast fungus [39]. Despite the large scale high-throughput screening and successful cloning of *AVR* genes, a complete understanding of them remains a challenge. Although comparative genomics data of field isolates are available, the exact diversity of the *AVR* locus is still unknown. Usually, after the outbreak of the disease epidemic, the resistant plants become susceptible and, in the following years, a new combination of natural variants in the blast fungus is observed. In Chinese monogenic lines, the resistance to rice blast fungus was introduced in 2000. In fact, five resistant genes were introduced, via the conventional breeding techniques, into the blast susceptible Japonica rice variety named Lijiangxin-tuan-heigu (LTH) [40,41]. At the protein level, *Pi9* is a member of the NBS-LRR genes that show broad-spectrum resistance to different rice blast strains.

In this study, we analyzed 326 *M. oryzae* isolates from the rice plants grown in Yunnan Province, China. We identified small variations in the *AVR* gene and its regulatory region. According to the gene-for-gene interaction model, the genetic variation in *AVR* may lead to a disruption of its corresponding *R* gene. The fungus-encoded *AVR* genes are targets of the plant-encoded *R* genes. It is clear that rice varieties harboring the *Pi9* gene show immunity to different rice blast strains. It is also well established that there is a strong correlation between *Pi9* and *AvrPi9.* Mechanistically, *Pi9* can recognize *AvrPi9*, which results in a broad-spectrum resistance to diverse rice blast isolates. The *AVR* genes evolve rapidly, resulting in the breakdown of their corresponding resistance genes. In a recent study on the natural variation of *AVR* genes in the Sichuan Basin of China, there was a loss of natural resistance against *M. oryzae* [42]. Therefore, it is believed that closed geographical monitoring of rice lines is important to control the spread of a virulent blast strain. In this study, we analyzed the spread of disease in the Yunnan province of China, where a monogenic resistant line containing the *Pi9* (IRBL9-W) *R* gene was cultivated on a large scale. We also included three non-rice hosts (wheat, ryegrass and *Setaria viridis*) to determine the natural variation of the *AVR-Pi9* genes. The complete sequencing of 328 isolates of *M. oryzae* was obtained. Interestingly, through sequence analysis, we identified the insertions, deletions and substitution mutations in the different isolates. These data led to the identification of seven unique haplotypes that were not previously known. The H1 haplotype was mostly identical to the existing blast strain sequences. The higher frequency of the H1 haplotype was due to its genetic identity with the existing strain in the coding region of the *AVR-Pi9* gene. 

The pathogenicity assay suggests that the resistance phenomenon is invalidated by the most prevalent H1 haplotype of *AVR-Pi9*. The mutations in the promoter region of the *AVR-Pi9* gene are intriguing. In the previous studies on AVR genes, it was reported that transposable element insertion sites are located in the promoter region [43,44]. The evolution of mutant magnaporthe races is due to the loss of avirulence genes, which have important interactions with the resistance genes. Even a small point mutation can cause the loss of avirulence. Previous study showed that the deletion of avirulence genes is a common phenomenon in diverse natural populations [45].

It is well established that the nucleotide insertions in *AVR* genes result in the loss of resistance [46]. Previous studies conducted on a large collection of different cultivars in China suggest that the *Pi9* locus in the rice genome confers broad spectrum resistance due to the presence of multiple novel alleles [47]. The allelic variation of the *Pi9* gene in the rice genome may be a strong reason for the presence of *AVR-Pi9* haplotypes [13]. It is possible that for each allele of *Pi9*, there is an evolution of *AVR-Pi9*. Previous studies suggest that there is a direct interaction from the *AVR-Pi9* gene with the LRR domain of its cognate *Pi9* gene [14]. Currently, more than one hundred *R* genes have been characterized, and several of them have been incorporated into rice varieties worldwide [13]. Most of these *R* genes contain nucleotide-binding site leucine-rich repeats (NBS-LRRs), which can potentially interact with pathogen *AVR* proteins [48]. However, the *R* genes *Pi-d2* and *Pi21* contain B lectin receptor kinase and proline-rich metal binding proteins, respectively, instead of NBS-LRRs [45]. In the future, it will be interesting to investigate whether the different haplotypes of *AVR-Pi9* are capable of interacting directly with their cognate *Pi9* gene.

## 5. Conclusions

We identified seven new haplotypes of the major *AVR-Pi9* gene from the field-grown rice population. Our findings suggest that there may be more variations in the AVR genes than expected. The diversity in AVR sequences can be alarming as the new haplotypes of the fungus can escape the resistance provided by the host genes. Due to the continuous arms race, the evolution of AVR genes may outpace the evolutionary rate of host resistance mechanisms. Therefore, in addition to genetic resistance, the management of fungal pathogens should also be practiced for sustainable crop production. 

## Figures and Tables

**Table 1:** Distribution of the *AVR-Pi9* genes and the avirulence isolates of the *M. oryzae* collected from Yunnan, China.

Host	Locations/Years	No. of Isolates	PCR Detection		Pathogenicity Assay ^a^	
			No. of Isolates with *AVR-Pi9*	Frequency (%)	No. of Avirulence Isolates	Frequency (%)
Rice	Northeastern/2012	44	44	100.0	44	100.0
	Central/2013	88	88	100.0	88	100.0
	Southeastern/2013	21	21	100.0	21	100.0
	Western/2013	127	127	100.0	122	96.1
	Northwestern/2014	21	21	100.0	21	100.0
	Southwestern/2014	25	25	100.0	25	100.0
	Total	326	326	100.0	321	98.5
Non-rice	Western/2014	1	1	100.0	1	100.0
	Central/2014	1	1	100.0	1	100.0
	Total	2	2	100.0	2	100.0

^a^ Pathogenicity test was performed on the resistant genotype (IRBL9-W) as described previously (35–38).

**Table 2:** Distribution of the *AVR-Pi9* haplotype in the different rice regions of Yunnan Province.

Haplotype	No. of Isolates	Frequency (%)	No. of Isolates and Frequency (%) in Each Region						Production ^a^	
			Northeastern	Central	Southeastern	Western	Northwestern	Southwestern	XI	GJ
H01	218	66.9	42 (95.5)	48 (54.5)	12 (57.1)	95 (74.8)	8 (38.1)	13 (52.0)	120 (69.4)	98 (64.1)
H02	87	26.7	1 (2.3)	30 (34.1)	9 (42.9)	25 (19.7)	13 (61.9)	9 (36.0)	43 (24.9)	44 (28.8)
H03	12	3.7	0	7 (8.0)	0	5 (3.9)	0	0	5 (2.9)	7 (4.6)
H04	2	0.6	0	0	0	0	0	2 (8.0)	2 (1.2)	0
H05	3	0.9	0	0	0	2 (1.6)	0	1 (4.0)	3 (1.7)	0
H06	1	0.3	1 (2.3)	0	0	0	0	0	0	1 (0.7)
H07	3	0.9	0	3 (3.4)	0	0	0	0	0	3 (2.0)
Total	326	100	44	88	21	127	21	25	173	153
No. of haplotypes			3	4	2	4	2	4	5	5
Index of diversity ^b^			0.088	0.579	0.490	0.400	0.472	0.592	0.456	0.505

^a^* XI* and *GJ* indicates Xian/Indica and Geng/Japonic, respectively. ^b^ Diversity index was calculated as the frequency of the haplotype types in the *M. oryzae* population following Fontaine’s method. Diversity index = (1 − ∑^n_i=1_^p_i_^2^) (where pi is the frequency of the haplotype i in a population).

**Table 3:** Haplotypes of the *AVR-Pi9* loci in the rice blast fungus of Yunnan, China.

Haplotype	No. of Isolates	Frequency (%)	Variant Locus ^a^									
			Promoter Region			CDS			Intron			
			Between 168–169	Between 812–827	912	1139	1169	1310	Between 1342–1343	1345	1374	1376
KM004023.1			-	CTCCTACACTGGGGCT	T	C	C	C	-	G	G	C
H1	218	66.5	.	.	.	.	.	.	-	.	.	.
H2	87	26.5	.	-	.	.	.	.	-	.	.	.
H3	12	3.7	T	-	.	.	.	.	-	.	.	.
H4	1	0.3	.	-	C	.	.	.	-	.	.	.
H5	4	1.2	T	.	.	.	.	.	-	.	.	.
H6	1	0.3	.	.	.	.	T	.	-	.	.	.
H7	3	0.9	.	.	.	.	.	.	-	.	.	T
H8 *	2	0.6	.	.	.	T	.	T	GCCCTGTACAATGCTTTTT	T	T	.
328	100											

^a^ “-” indicates absent or deletion. “.” indicates nucleotide identity with KM004023.1 (accession ID) from GenBank. * denotes the haplotypes isolated from non-rice hosts.

**Table 4:** The nucleotide identity of the *AVR-Pi9* isolated from the three non-rice hosts.

		Host and Isolate						
		Rice	Rice	Perennial Ryegrass	Perennial Ryegrass	Wheat	Wheat	*Setaria viridis* (L.) Beauv
Query	Size (bp)	P131	PY34	PgKY	PGPA	PY5033	PY6045	US71
AVR-Pi9_KM004023.1	3496 letters	2301/2302 (99%)	3490/3502 (99%), Gaps = 9/3502 (0%)	1242/1258 (98%), Gaps = 10/1258 (0%)	1242/1258 (98%), Gaps = 10/1258 (0%)	1237/1274 (97%), Gaps = 27/1274 (2%)	1237/1274 (97%), Gaps = 27/1274 (2%)	1372/1393 (98%), Gaps = 19/1393 (1%)

**Table 5:** Results from the Tajima’s neutrality test conducted for the *AVR-Pi9* in *M*. *oryzae* ^a^.

m	S	π	D
328	7	0.00006	−1.85719 *

^a^ The analysis involved 328 nucleotide sequences of *AVR-Pi9*. *S* indicates the number of segregating sites, *π* indicates nucleotide diversity, and *D* is the Tajima test statistic. * statistical significance and *p* < 0.05.

## Data Availability

Not applicable.

## Supplementary Materials

The following supporting information can be downloaded at: https://www.mdpi.com/article/10.3390/life13061320/s1, Figure S1. The haplotype-network-structured base on SNP (Panel-A) and the insert/deletion (Panel-B) of the eight *AVR-Pi9* alleles collected from rice and non-rice hosts. The original *AVR-Pi9* allele was designated as the H1 haplotype in the network. The dotted line in the network represents an extinct or a missing haplotype that has not been found among the samples. Each haplotype was separated by mutational events. All haplotypes are displayed as circles, and the circle size corresponds to the haplotype frequency. The H1 haplotype was the same as the *AVR-Pi9* obtained from GenBank (accession no. KM004023.1).
