# Supplementary material for: Emergence of Rice Blast AVR-Pi9 Resistance Breaking Haplotypes in Yunnan Province, China
Source: Life (Basel). 2023 Jun 4;13(6):1320. doi: 10.3390/life13061320 (PMC10302552; doi:10.3390/life13061320)
Supplement: Supplementary file 1 [file life-13-01320-s001.zip › life-2351150-supplementary.pdf]

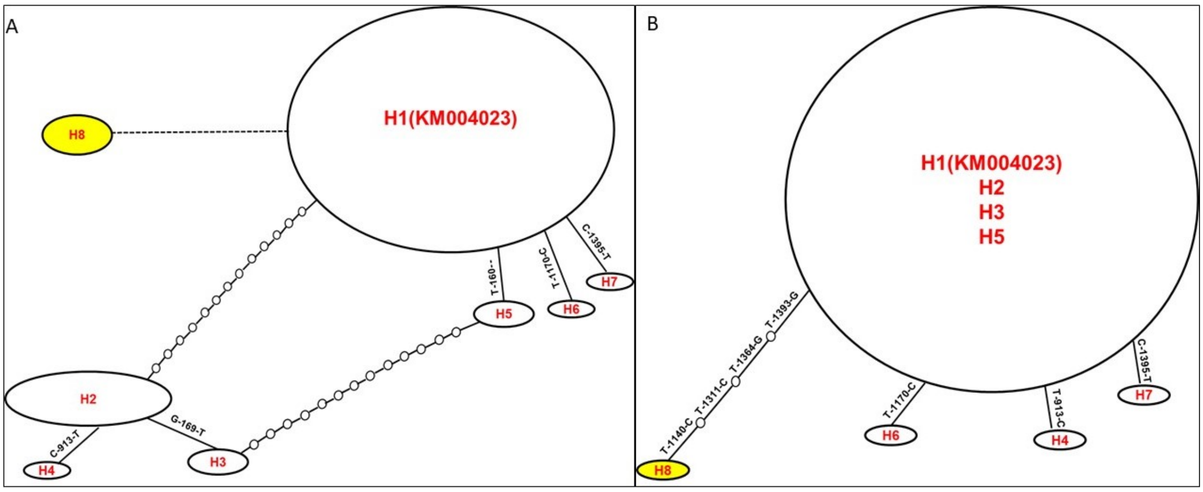

Figure S1. The haplotype network structured base on SNP (Panel-A) and insert/deletion (Panel-B) of the eight *AVR-Pi9* alleles collected from rice and non-rice host. The original *AVR-Pi9* allele was designated as the H1 haplotype in the network. The dotted line in the network represents an extinct or a missing haplotype not found among the samples. Each haplotype was separated by mutational events. All haplotypes were displayed as circles; circle size corresponds to the haplotype frequency. The H1 haplotype was the same as *AVR-Pi9* obtained from GenBank (accession no. KM004023.1).
